# Combined effects of physical activity and sedentary behavior on all-cause mortality in heart failure patients: A cohort study of national health and nutrition examination survey analysis

**DOI:** 10.3389/fcvm.2022.1027995

**Published:** 2022-10-14

**Authors:** Yunhui Zhu, Zhebin Chen, Songzan Chen, Guosheng Fu, Yao Wang

**Affiliations:** Key Laboratory of Cardiovascular Intervention and Regenerative Medicine of Zhejiang Province, Department of Cardiology, Sir Run Run Shaw Hospital, School of Medicine, Zhejiang University, Hangzhou, China

**Keywords:** physical activity, sedentary behavior, heart failure, mortality, NHANES

## Abstract

**Background:**

Physical activity and sedentary behavior are independently related to the risk of cardiovascular disease. Physical activity is recognized as having a protective effect, while being sedentary seems to be adverse. Nonetheless, the interactions between physical activity and sedentary behavior and the combined effect on the prognosis of heart failure patients remain unclear.

**Methods and results:**

This cohort study included 886 heart failure patients from the National Health and Nutrition Examination Survey (NHANES) 2007-2018. Physical activity and sedentary behavior were assessed by the NHANES questionnaires. The all-caused deaths of enrolled subjects were identified from National Death Index (NDI) database. During a median follow-up of 51 months, 321 (36.2%) deaths from any causes occurred. Multivariable Cox proportional hazards models were used to estimate the hazard ratios (HRs) and 95% confidence interval (CI) for the all-cause mortality in heart failure patients associated with physical activity and sedentary behavior. Physical activity was independently associated with lower mortality [HR = 0.51, 95% CI (0.38-0.68), *p* < 0.001] and sedentary behavior was associated with adverse prognosis [HR = 1.79, 95% CI (1.41–2.28), *p* < 0.001]. Kaplan–Meier survival curve showed that physical activity appeared to attenuate the negative consequences of SB, while sedentary behavior increased the all-cause mortality, particularly those without physical activity.

**Conclusion:**

Physical activity has a protective effect on HF patients’ prognosis, particularly those with sedentary behavior. Sedentary behavior independently exhibited a negative association in populations without physical activity, while it does not increase mortality in those with moderate physical activity.

## Introduction

Heart failure (HF) is a complicated syndrome at the end stage of various cardiovascular diseases (CVDs), and is a leading cause of morbidity and mortality (1). 50% of HF patients with reduced ejection fraction (HFrEF) die within 5 years from diagnosis (2).

Physical activity is considered a diagnostic and prognostic tool for chronic heart failure, as well as a therapeutic intervention of HF (3). In addition to being a primary prevention in heart failure (4), physical exercise is now a well-establish therapy for HF patients (5). European Society of Cardiology (ESC) guideline 2021 recommended that HF patients should perform proper exercise training (6). O’Connor et al. (7) showed that exercise training was associated with modest significant reductions in cardiovascular mortality or heart failure hospitalization after adjustment for highly prognostic predictors of the primary end point. Furthermore, exercise impairment is a crucial characteristic of HF, which is used as a prognostic factor in HF patients. Measured oxygen consumption (VO2) at peak physical activity is a widely used prognostic factor in HF (3). Other parameters related to physical exercise have also been shown to be strong predictors of mortality, including VO2 at the anaerobic threshold, O2 uptake efficiency slope, mean response time, and hemodynamic measurement during exercise (8).

In addition to the positive health effects of physical activity (PA), Sedentary behavior has been found to be associated with many health risks, including diabetes (9) and cardiovascular disease (10). Physical Activity Guidelines for Americans in 2018 stressed the importance of reducing sedentary time for both general and cardiovascular health (11). It was found that sedentary behavior may adversely affect cardiovascular metabolism (12), resulting in declining quality of life and increased risk of morbidity and mortality (13). In particular, Deborah Rohm Young et al. (14) found that longer sedentary time was correlated to a higher incidence of heart failure in men aged ≥ 45 years. Moreover, patients with HF exhibit a longer sedentary behavior (15), which may further contribute to severe disability and higher mortality (16, 17). However, previous studies demonstrated more evidence that physical inactivity and sedentary time were associated with new-onset HF (18). The effect of sedentary behavior on outcomes in patients with established HF, especially HFpEF, is not well studied.

Here, it is necessary to emphasize the difference between sedentary behavior and physical inactivity. Although sedentary behavior and physical activity are on opposite sides of the energy consumption (19), and the previous studies (20, 21) have tended to treat sedentary behavior as a specific classification in the intensity of physical activities, sedentary behavior is likely to be considered an independent risk factor for cardiovascular health outcomes (22). In other words, it is reasonable that a patient engages in moderate or vigorous-intensity physical activity while sitting for several hours a day. Therefore, it is essential to understand the interactions between sedentary behavior and physical activity on health outcomes. To determine the prognostic role of physical activity and sedentary behavior in HF patients, this study investigated the combination relationship between PA/SB and all caused mortality in patients with established HF from the NHANES data.

## Materials and methods

### Study population

**FIGURE 1:**
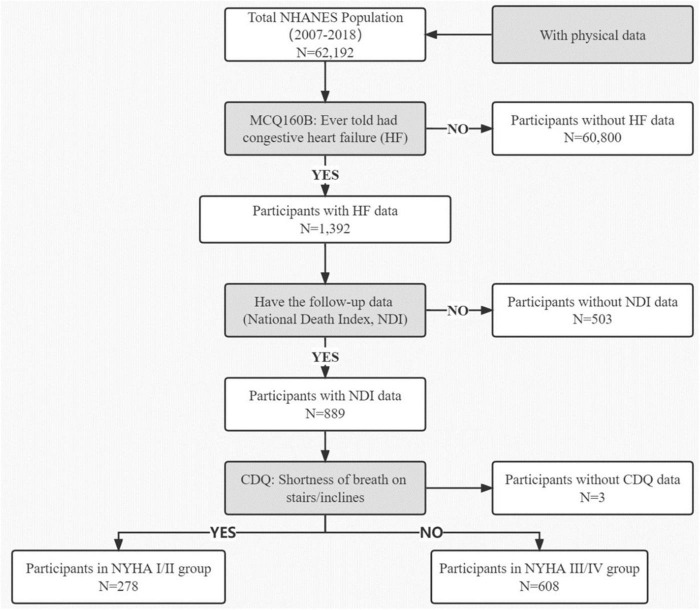
Study flowchart. NHANES, National Health and Nutrition Examination Survey; HF, Heart failure; NDI, National Death Index; CQD, Cardiovascular Health Questionnaire; NYHA, New York Heart Association Classification of Heart Failure.

This study included data from the National Health and Nutrition Examination Survey (NHANES) 2007–2018. The NHANES database is an ongoing cross-sectional survey conducted by the National Center for Health Statistics (NCHS). The survey selects approximately 10,000 participants nationwide every 2-year cycle by stratified random sampling, and then collects and incorporates their interviews, physical examinations, and laboratory tests. Moreover, the follow-up data is from National Death Index (NDI). The flow chart of the current study is shown in Figure 1. A total of 886 subjects diagnosed with congestive heart failure were enrolled. The inclusion criteria: (1) participants with self-reported a Congestive Heart Failure diagnosis; (2) participants with NDI data; (3) participants with Cardiovascular Health Questionnaire (CDQ) data which classifies patients into two groups according to the New York Heart Association (NYHA) Classification of Heart Failure. Institutional Review Board approval was not required as the NHANES represents an adequately de-identified and publicly available dataset (23).

### Physical activity and sedentary behavior assessment

Detailed information about physical performance status and sedentary time was assessed by the NHANES physical activity questionnaire. The type, frequency, and duration of leisure-time physical activity were collected. According to Ainsworth’s Compendium of Physical Activity, the intensity of physical activities was represented by the metabolic equivalent of task (MET) (24), which is the objective measure of the ratio of the rate at which a person expends energy. 1 MET is roughly equal to the energy expenditure in a quiet and seated position, defined as 1 kcal/(kg*hour) or 3.5 mL of consumed oxygen/(kg*minute). World Health Organization (WHO) recommends a minimum of weekly physical activity of 7.5 METs-hour in general population (25). The NHANES physical activity questionnaire recorded and collected the activity time per week for each participant. They were divided into active group and non-active group based on converted METs. We defined 0 MET-hour/week as non-PA and ≥ 0.1 MET-hour/week as PA. The sedentary activity in the NHANES questionnaire includes sitting at work, at home, in a car or bus, reading, watching television, or using a computer, and notably, do not include time spent sleeping. Sitting for more than 6 h a day is defined as sedentary behavior (22).

### Study outcomes and follow-up

The primary outcome of this study was the overall mortality in heart failure population from National Death Index (NDI) database. Patients with heart failure who were enrolled were followed up every year to assess their health status.

### Statistical analysis

Data were described as count (%) for categorical variables and mean ± standard deviation for continuous variables. Sampling weights were used in all analyses to generalize results to the United States civilian non-institutionalized resident population. Baseline characteristics were compared by χ^2^ test or Kruskal–Wallis test. Univariate Cox regression was performed for every exposure variable to predict the overall mortality. A Multivariate Cox proportional hazard model was used to analyze the association between physical activity, sedentary behavior, and overall mortality after adjusting age, marital status, and BMI. Kaplan–Meier plots were generated to assess the event-free survival. The difference between groups was compared by log-rank test. All missing data were excluded along the analytical process. All analyses and plots were conducted with R statistical software version 4.1.2. *P* < 0.05 was considered statistically significant. All the raw codes and data were provided in the Supplementary Data File named Rawcode.zip.

## Results

### Baseline characteristics

During the identified period of study, a total of 1,392 HF patients enrolled from NHANES. However, 506 subjects were excluded due to the missing data of NDI or CDQ. Of the 886 participants, 278 subjects (31.4%) were assessed as NYHA I/II, and 608 subjects (68.6%) were assessed as NYHA III/IV. The baseline descriptive characteristics for the study population of two groups are presented in Table 1. Overall, 80% were over 60 years, 54.7% were men, 52.8% were non-Hispanic White, 60.6% had more than high school education, 47.5% were married, 21.6% had a poverty index ratio of more than 3, 79.7% had a BMI more than 25, 58.8% were current smoking. Compared with patients in NYHA I/II group, patients in NYHA III/IV group were more likely to be younger than 60 years (33.0% vs. 13.7%, *p* = 0.001), more non-Hispanic White (56.2% vs. 45.3%, *p* = 0.013), higher BMI above 30 (54.2% vs. 43.7%, *p* = 0.005) and more smoking (62.7% vs. 50.4%, *p* = 0.001).

**TABLE 1:** Demographic characteristics of heart failure participants (*N* = 886, weighted count 3,959,970).

Descriptive variables	Overall (%)	NYHA I/II group (%)	NYHA III/IV group (%)	*P*
General Population	886	278	608	
Age				0.001
20–59 years	177 (20.0)	37 (13.7)	140 (33.0)	
60–85 years	709 (80.0)	241 (86.7)	468 (77.0)	
Gender				0.159
Male	485 (54.7)	142 (51.1)	343 (56.7)	
Female	401 (45.3)	136 (48.9)	265 (43.6)	
Race-Ethnicity				0.013
Mexican American	78 (8.8)	35 (12.6)	43 (7.1)	
Other Hispanic	65 (7.3)	21 (7.6)	44 (7.2)	
Non-Hispanic White	468 (52.8)	126 (45.3)	342 (56.2)	
Non-Hispanic Black	237 (26.7)	82 (29.5)	155 (25.5)	
Other Race	38 (4.3)	14 (5.0)	24 (3.9)	
Education Level				0.079
Less than 11th Grade	348 (39.4)	121 (43.5)	227 (37.5)	
High school	223 (25.2)	55 (19.8)	168 (27.7)	
College	215 (24.3)	71 (25.5)	144 (23.8)	
College Graduate	98 (11.1)	31 (11.2)	67 (11.1)	
Marital status				0.465
Married	421 (47.5)	134 (48.2)	287 (47.2)	
Living with partner	17 (1.9)	3 (1.1)	14 (2.3)	
Other	448 (50.6)	141 (50.7)	307 (50.5)	
Poverty index ratio				0.643
Less than 1	211 (26.5)	65 (26.4)	146 (26.5)	
1–3	413 (51.9)	123 (50.0)	290 (52.7)	
3 and above	172 (21.6)	58 (23.6)	114 (20.7)	
BMI (kg/m^2^)				0.005
Less than 25	169 (20.2)	66 (26.2)	103 (17.7)	
25–30	240 (28.7)	76 (30.2)	164 (28.1)	
30 and above	426 (51.0)	110 (43.7)	316 (54.2)	
Smoking				0.001
Yes	521 (58.8)	140 (50.4)	381 (62.7)	
No	365 (41.2)	138 (49.6)	227 (37.3)	

Data are expressed as n (% cohort).

NYHA: New York Heart Association Classification of Heart Failure; BMI: Body mass index.

### Main outcomes

There were 321 (36.2%) deaths from any causes during a median follow-up of 51 months. Univariable Cox regression analysis was shown in Table 2. The results revealed that age ≥ 60 (*p* < 0.001), unmarried status (*p* < 0.001) and sedentary behavior (*p* < 0.001) were found to be significant risk factors for the all-cause mortality. Conversely, BMI > 30 (*p* < 0.001), and physical activity (*p* < 0.001) could be considered as the indicators of reduced risk of death in heart failure population.

**TABLE 2:** Univariate cox regression factors associated with all caused death.

Descriptive variables	HR (95% CI)	*P*
**Age**		
20–59 years	Reference	
60–85 years	2.01 (1.43–2.83)	< 0.001
**Gender**		
Male	Reference	
Female	1.01 (0.81–1.26)	0.9
**Race-ethnicity**		
Mexican American	1.03 (0.71–1.51)	0.86
Other Hispanic	0.47 (0.28–0.79)	0.005
Non-Hispanic White	Reference	
Non-Hispanic Black	0.66 (0.51–0.87)	0.003
Other Race	0.96 (0.57–1.62)	0.88
**Education level**		
Less than 11th Grade	Reference	
High school	1.14 (0.87–1.5)	0.35
College	0.79 (0.58–1.06)	0.12
College Graduate	1.03 (0.72–1.49)	0.86
**Marital status**		
Married	Reference	
Living with partner	0.66 (0.24–1.78)	0.41
Other	1.52 (1.21–1.9)	< 0.001
**Poverty index ratio**		
Less than 1	Reference	
1–3	1.22 (0.92–1.62)	0.16
3 and above	0.89 (0.62–1.27)	0.52
**Body mass index (kg/m^2^)**		
Less than 25	Reference	
25–30	0.53 (0.39–0.73)	< 0.001
30 and above	0.5 (0.38–0.66)	< 0.001
**Smoking**		
Yes	Reference	
No	1.07 (0.86–1.34)	0.54
**NYHA**		
NYHA I/II	Reference	
NYHA III/IV	1.17 (0.99–1.38)	0.07
**Physical activity**		
0 MET	Reference	
≥ 0.1 MET	0.41 (0.31–0.55)	< 0.001
**Sedentary behavior**		
No	Reference	
Yes	2.02 (1.61–2.53)	< 0.001

HR, Hazard ratio; CI, confidence interval; NYHA, New York Heart Association Classification of Heart Failure; MET, metabolic equivalent of task.

**FIGURE 2:**
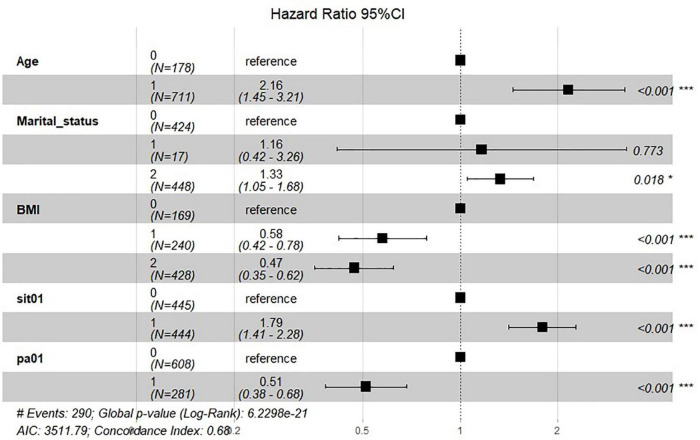
Forest-plot for multivariate Cox regression associated with all caused death. Age = “0” represents 20–59 years, “1” represents 60–85 years. Marital status = “0” represents married, “1” represents living with partner, and “2” represents others. BMI = “0” represents BMI < 25, “1” represents 20–25, and “2” represents BMI ≥ 25. Sit01 = “0” represents no sedentary behavior while “1” represents group with sedentary behavior. Pa01 = “0” represents no physical activity while “1” represents group with physical activity. *P < 0.05; ***P < 0.001.

Furthermore, Figure 2 shows the results of multivariate Cox regression analysis including above variables. Age ≥ 60 [HR = 2.16, 95% CI (1.45–3.21), *p* < 0.001], unmarried status [HR = 1.33, 95% CI (1.05–1.68), *p* = 0.018], BMI > 30 [HR = 0.47, 95% CI (0.35–0.62), *p* < 0.001], sedentary behavior [HR = 1.79, 95% CI (1.41–2.28), *p* < 0.001] and physical activity [HR = 0.51, 95% CI (0.38–0.68), *p* < 0.001] were all independently related to risk for all caused death in HF population. This indicated a protective effect of physical activity and the adverse effect of sedentary behavior on the prognosis of heart failure patients.

### Association of PA and SB with mortality in two NYHA groups

**FIGURE 3:**
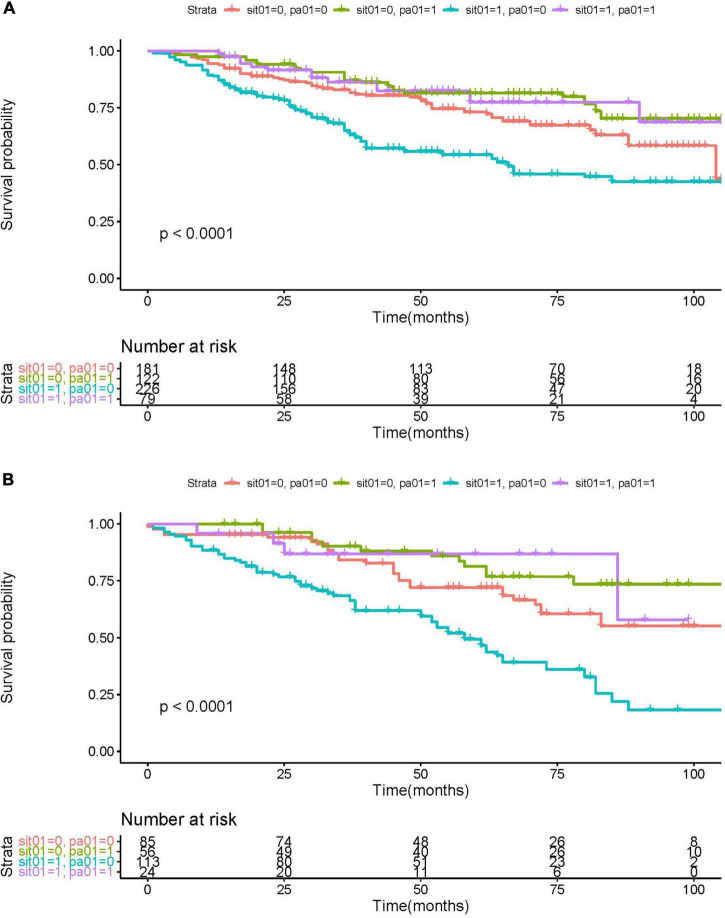
KM survival curve for NYHA I/II group **(A)** and NYHA III/IV **(B)**, sorted by physical activity (PA) and sedentary behavior (SB). Red curve: without PA or SB (“sit01” = 0, “pa01” = 0); green curve: PA without SB (“sit01” = 0, “pa01” = 1); blue curve: SB without PA (“sit01” = 1, “pa01” = 0); purple curve: PA with SB (“sit01” = 1, “pa01” = 1).

Since exercise impairment and sitting for a longer time are critical characteristics of HF, especially in patients with worse cardiac function, HF patients were divided into NYHA I/II group and NYHA III/IV group. Kaplan–Meier survival curve for different combinations of physical activity and sedentary behavior groups was shown in Figure 3. In both NYHA I/II and NYHA III/IV groups, the population with sedentary behavior and physical inactivity had the lowest survival-free probability (blue curve). There was no significant difference in the prognosis of HF patients with physical activity, whether they were with sedentary behavior (purple curve) or not (green curve). The results indicated that high levels of physical activity seem to attenuate the negative effects of SB. Sedentary behavior increases the risk of all-cause mortality in HF patients, particularly those without physical activity. On the contrary, this adverse prognostic effect of sedentary behavior is not significant when the HF patient had some exercise.

## Discussion

In this retrospective study, we enrolled 886 patients with established heart failure in NHANES 2007–2018 data. We provided evidence that the prognosis of HF is closely associated with physical activity and sedentary behavior independently. In particular, sedentary behavior could independently predict higher overall mortality along with the following up. Furthermore, SB exhibited more adverse effects in populations without physical activity, while in those with moderate physical activity, SB does not increase mortality. Similarly, PA could attenuate the negative impact of SB in HF patients. However, the beneficial effect of physical activity was not significant when patients were not with SB.

A series of preclinical studies suggested the potential mechanisms for the association between sedentary behavior and adverse cardiovascular outcomes. Bey et al. (26) used hindlimb suspension acutely preventing ambulatory activity in a rat model to mimic sedentary behavior in humans. They found that lipoprotein lipase (LPL) activity was suppressed in skeletal muscle, causing decreased muscle uptake of plasma triglyceride and high-density lipoprotein cholesterol (HDL-C) concentration. Furthermore, a global gene-expression profiling study using Affymetrix microarrays was conducted to compare mRNA levels in the soleus muscle in rats under hindlimb unloading. Bey et al. (27) identified 38 genes most sensitive to loading/activity in rat skeletal muscle involved in protein synthesis/degradation and energy metabolism. Besides, several studies showed that sedentary behavior likely reduced the expression of key metabolic regulators. Joseph et al. (28) found that NAD-dependent deacetylase SIRT3 (sirtuin-3) was lower within the skeletal muscle cells in sedentary individuals, leading to decreased expression of PGC-1α, a key regulator of mitochondrial mass/function. On the contrary, suspending sedentary time with light-intensity physical activity can increase the expression of nicotinamide *N-*methyltransferase, a modulator in anti-inflammatory pathway (12). Overall, sedentary behavior can alter metabolism at the level of skeletal muscle resulting in gross metabolic disturbances, which indicate some potential mechanisms for the sedentary activity to reduce cardiovascular prognosis. More evidence is required to determine the pathophysiological pathways of sedentary behavior and whether these pathways differ from those associated with physical activity.

In contrast to sedentary behavior, the benefits of physical activity on heart failure are multifaceted. Kemi et al. (29) revealed that physical activity, especially aerobic interval training, enhances cardiomyocyte contractility and faster reuptake of cytoplasmic calcium by phosphorylation of threonine-17 residue. Animal models have provided evidence that exercise training enhances calcium handling *via* calcium-transporting ATPase on the sarcoendoplasmic reticulum (30) and reduces circulating inflammation markers, including C-reactive protein. This protects cells from inflammation-mediated myocardial fibrosis and dysfunction (31). At the level of mRNA, physical activity has been shown to increase the expression of SIRT3 (32) and PGC-1α (33), which regulate the early processes of mitochondrial biogenesis, while decreasing the expression of catabolic mRNA in skeletal muscle such as FOXO3a (forkhead box O3), MuRF-1 (muscle RING-finger protein-1) and atrogin-1 (34). Moreover, the lifelong exercise training has been proved to prevent decrements in compliance and distensibility (35), reduce stiffness and afterload, and thus maintain cardiac function.

Following the basic research studies described above, a large number of clinical research on the associations between SB or PA with cardiovascular outcomes were conducted. The evidence of the associations between physical activity (11) or sedentary behavior (36) with mortality from CVD is solid and is consistent with our study. However, previous studies about the interaction between sedentary behavior and physical activity were insufficient, and joint effects on outcome were unclear. Ekelund et al. (37) investigated the combined effects of SB and PA on mortality from CVD and all causes and demonstrated the role of SB across different activity levels, which is parallel to our results, with the difference that we focused on mortality in heart failure patients. Moreover, compared to previous studies, our study found consistent findings in HF populations with different NYHA classes, corresponding to different exercise tolerance. On the other hand, our study was a community-based study rather than a hospital-based study. The characteristics of HF patients are completely different from hospital to community settings, where HF patients are always in stable condition. Therefore, the results obtained from the NHANES database are adaptable to explore SB and PA.

### Limitations

The potential limitation of our present study should be clarified. Firstly, as with any cohort study, the study cannot extrapolate the causal relationship between PA or SB and the prognosis of HF patients. However, the value of higher physical activity and less sedentary behavior as an independent beneficial predictor can be determined. Secondly, the endpoint data from the NDI database selected only all-cause mortality, without further differentiation of the causes of death in patients with heart failure, such as cardiovascular mortality. Thirdly, in terms of variable classification, a distinction is made between the presence or absence of PA and SB, without the stratified level of intensity and duration. Therefore, it is unfortunate that we could not obtain the dose-dependent effect of PA and SB on mortality in the HF population. Last, due to the characteristics of the NHANES database, we could not obtain several detailed variables, such as NT-proBNP and LVEF, which are associated with mortality in heart failure patients but not included in NHANES. Furthermore, some other determinants of functional limitation and sedentary behavior, such as fractures and obesity, were not analyzed separately. Focusing on these determinants as clinical indicators may be beneficial for improving the prognosis of heart failure.

## Conclusion

Physical activity has a protective effect on HF patients’ prognosis, while sedentary behavior independently plays an adverse role. Physical activity could attenuate the negative effect of sedentary while this beneficial effect was insignificant in patients without sedentary. Analogously, sedentary behavior exhibited a negative association in populations without physical activity, while in those with moderate physical activity, SB does not increase mortality.

## Data availability statement

The datasets presented in this study can be found in online repositories. The names of the repository/repositories and accession number(s) can be found in the article/supplementary material.

## Ethics statement

Institutional Review Board approval was not required as the NHANES represents an adequately de-identified and publicly available dataset. The patients/participants provided their written informed consent to participate in this study.

## Author contributions

YW and GF conceived and designed the study. YZ organized these data and drafted the manuscript with the help of ZC and SC. YZ and ZC analyzed the data and drew the pictures. YW and SC reviewed and edited the manuscript. YW and GF detected any errors in the whole process. All authors have read and approved the manuscript for submission.

## Abbreviations

BMI: Body Mass Index
CI: confidence interval
CVD: Cardiovascular Diseases
CQD: Cardiovascular Health Questionnaire
ESC: European Society of Cardiology
FOXO3a: Forkhead box O3
HF: Heart Failure
HFrEF: Heart Failures with reduced Ejection Fraction
HR: Hazard ratio
LPL: Lipoprotein Lipase
MET: Metabolic Equivalent of Task
MuRF-1: Muscle RING-Finger protein-1
NCHS: National Center for Health Statistics
NDI: National Death Index
NHANES: National Health and Nutrition Examination Survey
NHYA: New York Heart Association
PA: Physical Activity
PGC-1α: Proliferator-activated receptor γ Coactivator 1-α
SB: Sedentary Behavior
SIRT3: Sirtuin-3.
